# Evaluation of stretch reflex synergies in the upper limb using principal component analysis (PCA)

**DOI:** 10.1371/journal.pone.0292807

**Published:** 2023-10-12

**Authors:** Frida Torell

**Affiliations:** Physiology Section, Department of Integrative Medical Biology, Umeå University, Umeå, Sweden; Opole University of Technology: Politechnika Opolska, POLAND

## Abstract

The dynamic nature of movement and muscle activation emphasizes the importance of a sound experimental design. To ensure that an experiment determines what we intend, the design must be carefully evaluated. Before analyzing data, it is imperative to limit the number of outliers, biases, and skewness. In the present study, a simple center-out experiment was performed by 16 healthy volunteers. The experiment included three load conditions, two preparatory delays, two perturbations, and four targets placed along a diagonal path on a 2D plane. While the participants performed the tasks, the activity of seven arm muscles were monitored using surface electromyography (EMG). Principal component analysis (PCA) was used to evaluate the study design, identify muscle synergies, and assess the effects of individual quirks. With PCA, we can identify the trials that trigger stretch reflexes and pinpoint muscle synergies. The posterior deltoid, triceps long head, and brachioradialis were engaged when targets were in the direction of muscle shortening and the perturbation was applied in the opposite direction. Similarly, the pectoralis and anterior deltoid were engaged when the targets were in the direction of muscle shortening and the perturbation was applied in the opposite direction. The stretch reflexes were not triggered when the perturbation brought the hand in the direction of, or into the target, except if the muscle was pre-loaded. The use of PCA was also proven valuable when evaluating participant performance. While individual quirks are to be expected, failure to perform trials as expected can adversely affect the study results.

## Introduction

Muscles must collaborate to produce voluntary movement. Surface electromyography (EMG) electrodes can be placed on the surface of the skin to record muscle activity. For the upper extremities the pattern of muscle activation, when working in a diagonal 2D-plane of movement, involves an internal rotation of the shoulder and extension and flexion of both the elbow and the shoulder joints. It has been shown using surface EMG, that the EMG activity matches what would be expected based on physiology. Meaning that that elbow extension activates e.g., triceps long head, while elbow flexion activates e.g., brachioradialis and biceps [1–3]. Both elbow flexion and elbow extension activate the triceps lateral head [1]. Shoulder flexion activates e.g., anterior deltoid, and pectoralis (clavicular fibers), while shoulder extension activates e.g., posterior deltoid and triceps long head [4]. Internal rotation of the shoulder activates e.g., anterior deltoid and pectoralis [5, 6].

Robotic assessment tools are able to record high-dimensional kinematic data from the upper limbs of a participant with a high degree of reliability [7–9]. One of the available robotic platforms is Kinarm [10, 11]. Since the startup of the Kinarm lab the robotics platforms have been used to provide objective parameters of motor, sensory, and cognitive function using the participants’ arms. The Kinarm is able to trigger stretch reflex responses by allowing the participant to experience haptic perturbations [12]. By placing multiple surface EMG electrodes on different muscles, it is possible to study the stretch reflexes in active reaching tasks. The stretch reflex is a fundamental part of motor function and pivotal part of coping with environmental changes when we are experiencing disturbances of the planned trajectory. The sensitivity of the stretch reflex must adapt to the complexity of the movement and the switch between movement and posture. The sensitivity of the stretch reflex can be used to assess peripheral neural processing and shed light on the centuries old question about how movement is initiated. Using the Kinarm platform it has been found that the reflex responses can be modulated subconsciously to accommodate the execution of voluntary movements. For example, active reaching tasks have shown that the stretch reflex modulation occurs depending on target shape [13], obstacles [14], static and moving targets [15], and target cue direction [16], for review see Scott, 2016 [17]. Overall the Kinarm platforms have helped moving the field away from the schoolbook simplifications that the fusimotor neurons are only a part of alfa-gamma coactivation [18, 19], towards a more nuanced view where the independence of the fusimotor system is acknowledged [20]. However, the debate is still ongoing on the impact of this independence [21–24].

To shed light on some of the remaining questions a baseline of data inspection and pre-evaluation is needed. By recording the muscle activity using multiple surface EMG electrodes on different muscles, it is possible to identify common activity patterns of different muscles. For this purpose of analyzing data from several different, simultaneously recorded, surface EMGs, principal component analysis (PCA) is the favorite choice owing to its simplicity, interpretability, and availability [25, 26]. PCA is an unsupervised method that is used to reduce dimensionality and visualize groupings, common trends as well as outliers. PCA displays the projected data into principal components representing the directions of maximum variance with the constraint that they have to be perpendicular [27]. Naik *et al*. (2016) have written a comprehensive review on how PCA can be used to decompose surface EMG data into motor unit action potentials (MUAPs) [26]. The list of studies of surface EMG signals using PCA is very long. The purposes of previous studies have for example been to identify muscle groupings across movement types e.g., Bengoetxea *et al*. 2014 [28], identify and classify upper limb motions e.g., Veer & Vig [29], perform channel selection and improve the accuracy of the estimated force e.g., Hajian *et al*. [30], use PCA as a filtering step to improve the output of neural classification methods e.g., Merzoug *et al*. [31], gesture identification Qi *et al*. [32], improve the function of prosthetics [33], and to study inter- and intra-muscular synergies in finger muscles [34]. Daffertshofer *et al*. have written a tutorial on how to apply PCA to EMG data and kinematics data [35]. In this way, PCA is a simple yet powerful tool in the multivariate toolbox, when working with EMG data.

PCA also has the potential to be used to evaluate study designs when working with stretch reflex experiments. Two of the greatest challenges when working with surface EMG data are that the data are noisy and there are between and within individual differences in muscle activation. The noisiness of the data is often overcome using different normalization and filtering techniques, smoothing strategies and by recording multiple repetitions. The between participant differences on the other hand are often overlooked. They are presumed to be representative, and their individual variation is not displayed, nor accounted for. One common strategy is to present one or two examples and/or to present a population mean value of the observed EMG curves with some error bars. To ensure that the data are not just influenced by a few strong outliers a PCA based pre-evaluation is suggested in this paper. The method suggested here can be used both to ensure that the study design triggers the stretch reflexes in the intended way and that the participants display coherent EMG profiles. In other words, this is a way to ensure that the participants have performed the task according to what has been expected of them. Proper performance must be ensured, and the degree of individual variation must be understood before averaging the data to obtain consensus EMG profiles that intend to shed light on any research question.

## Materials and methods

In the present study, PCA was used to evaluate the muscle activity of seven upper limb muscles during a center-out task, which involved elbow and shoulder flexion and extension. The experiment involved a simple computer game with four potential targets, placed along a diagonal path on a 2D plane. The starting position was referred to as the ’origin’ and to reach two of the targets the participant had to push the handle (requiring increased internal rotation of the shoulder, shoulder flexion, and elbow extension), while the other two targets were reached by pulling the handle (shoulder extension and elbow flexion). The experimental design also explored three load conditions, two preparatory delays, and two perturbations (applied along the same diagonal path).

### Subjects

In the current study, a delayed-reach experiment was performed by 16 neurologically healthy participants recruited from 15^th^ of October 2020 until 11^th^ of August 2021. The participants were collected using accindi.se. There were six inclusion criteria that were used. Each participant had to (1) be neurologically health, (2) have normal movement range, (3) be 18–40 years old, (4) be 165–185 cm tall, (5) be right-handed (defined as writing with their right hand) and (6) have a Swedish social security number to be covered by our insurance. The gender distribution was equal (i.e., there were eight male and eight female) and the mean age of 26.9 ± 5.4 years (average age ± standard deviation). All sixteen participants were naïve to the task, prior to participation. All participants gave informed, written consent and were informed that they could terminate the trial at any time, per the Declaration of Helsinki. None of the participants chose to end their participation. After the experiment, the participants were financially compensated for their contribution. This experiment was a part of a research program approved by the Ethics Committee of Umeå University, Umeå, Sweden.

### Robotic platform

**Fig 1:**
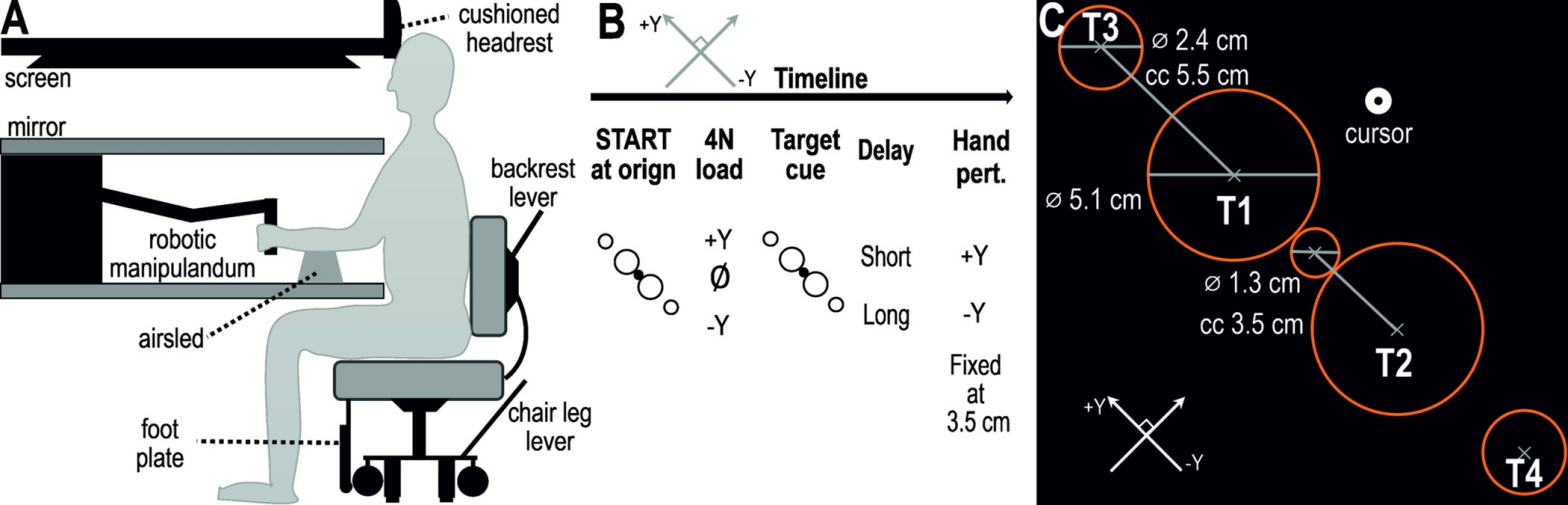
The robotic platform and experimental setup. **(A)** The participant manipulated the position of a robotic handle while playing a simple computer game with four potential targets situated along the Y-dimension. Throughout the experiment, the participants sat in an adjustable chair and had their right arm secured to the handle. All visual stimuli were projected onto a one-way mirror. The participants could not view their arm or the robotic handle, instead, the position of their hand was represented by a cursor (see Materials and Methods for more details). **(B)** Each new trial was initiated when the cursor was brought to the origin and stayed there for 1–1.5 seconds. This action initiated the load condition of the particular trial (no load or a slow-raising 4N load in either the +Y or -Y direction). Regardless of load condition, the participant must remain immobile inside the origin. A target was cued by turning red and remained cued either for 250 ms or 750 ms. This preparatory delay was followed by a rapid position-controlled perturbation of the hand (3.5 cm in 150 ms, during which the cursor position was frozen). At the end of the perturbation, the cued target turned green (the ’Go’ signal), and the participant had to move the cursor to the target as straight and swiftly as possible. **(C)** The continuously displayed potential targets. Origin diameter 1.3 cm. Near targets were spaced 3.5 cm from the center of the origin and had a diameter of 5.1 cm. The far targets were placed 9 cm from the center of the origin and had a diameter of 2.4 cm. Abbreviations: T3 = target 3, T1 = target 1, T2 = target 2, T4 = target 4, cc = center-to-center distance.

Throughout the experiment, the participants sat in an adjustable chair, in front of the Kinarm robotic platform (Kinarm end-point robot, BKIN Technologies Ltd., Canada), with their right hand grasping the right handle of the robotic manipulandum, see Fig 1. The adjustable chair was first adjusted to accommodate the participant’s height. The participants were seated with their knees at a 90° angle. The chair has an adjustable foot plate where shorter participants could rest their feet if the desired angel was not achieved when their feet were resting on the ground. Wheels allow the chair to be pushed as close to the table as possible. A D-shaped recess in the tabletop secures the participants torso in place. Once the chair was in place, a lever was pushed to release four metal legs that stabilize the chair throughout the experiment. The participants rest their head against a couched, height adjustable plate, further stabilizing the posture and reducing fatigue in the neck area. The right forearm was then placed in a customized foam structure, referred to as the ’glove’, resting on an airsled. Each trial started at the origin, where the starting position involved internal rotation of the shoulder, the elbow at approximately 90° angle, and the forearm in mid-rotation (thumb pointing up). A perturbation in the +Y direction (as defined in Fig 1) increased the internal rotation of the shoulder and caused flexion of the shoulder and extension of the elbow. A perturbation in the–Y direction caused an extension of the shoulder and flexion of the elbow. The airsled was supplied by compressed air. In combination with the tabletop material this allowed frictionless movement of the arm in a 2D plane. The mechanical connection between the glove, forearm, and handle was secured using leather fabric with Velcro attachments. In this way, the glove ensured that the wrist was in straight alignment with the forearm and kept the forearm in mid-rotation throughout the experiment. The Kinarm robotic platform measured kinematic data throughout the experiment, while simultaneously recording forces exerted by the participant using sensors inside the handle (Six-axis force transducer; Mini40-R, ATI Industrial Automation, U.S.A.). The kinematic data that were extracted included position on the 2D plane (*x*, *y*), 2D acceleration (*x*acc, *y*acc), 2D velocities (*x*vel, *y*vel), 3D forces (*x*, *y*, *z*), and 3D torques (*x*, *y*, *z*). A sampling rate of 1kHz was used to record position and force data.

### Experimental design

The participants viewed the content of a screen projected onto a one-way mirror (Fig 1A). In this way, the participants could not view the studied hand, as it was under the one-way mirror. Instead, the position of their hand was represented by a white dot (’cursor’; 1 cm diameter). This experiment included four potential targets, two ’near’ targets and two ’far’ targets, situated along the Y dimension. The ’origin’ circle outline was 1.3 cm in diameter. The center-to-center distance between the origin and the two ’near’ targets (circles; 5.1 cm diameter) was 3.5 cm and 9 cm for the ’far’ targets (circles; 2.4 cm diameter). Participants were continuously shown the orange circle outlines that depict all potential targets (Fig 1C).

A new trial began when the participant brought the cursor inside the origin and remained immobile for a random period of 1–1.5 seconds. The robotic arm then generated the load condition that was involved in this particular trial. The trial either involved a slow-rising 4N load applied in the +Y or -Y direction or no such pre-load was applied (Fig 1B). Regardless of load condition, the rise-time was 800 ms and the hold-time was 1200 ms. Provided that the cursor had remained immobile inside the origin during the rise- and hold-time, one of the potential targets was cued by turning red. Indicating that this is where the participant will be requested to move the cursor next. The cursor had to continue to remain immobile inside the origin for a ’preparatory period’ of 250 ms or 750 ms. The preparatory period was followed by a position-controlled mechanical perturbation applied in either the +Y or -Y direction (3.5 cm displacement, 150 ms rise-time, and no hold), during which the cursor position was frozen. The position-controlled mechanical perturbation was designed to stimulate ’naturalistic’ movement with an approximate bell-shaped velocity profile. To ensure appropriate hand kinematics on each trial, the Kinarm platform was allowed to exert a suitable stiffness maximum ~40,000 N/m), regardless of load conditions. At the end of the perturbation, the cued red target turned green (the ’Go’ signal), and the participant had to move the cursor inside the target as straight and swiftly as possible.

Once the participant had reached the target, they had to remain immobile inside the target borders for 300 ms. They then received visual feedback on how they had performed on this particular trial. This feedback was based on how quickly they had reached the target, once the cued target turned green (i.e., after the ’Go’ signal). If >1200 ms elapsed they received the visual feedback ’Too Slow’, and if they reached the target after 400–1200 ms they received the visual feedback ’Correct’. There was also a time limit of <400 ms, resulting in the feedback ’Too fast’. Regardless of the message, the visual feedback was displayed for 300 ms. Thereafter, the participants returned the cursor inside the origin to initiate the subsequent trial. If the participant decided to take a break they could move the cursor to the side, instead of returning it to the origin. However, brakes were normally encouraged between blocks and normally lasted < 5 minutes. One ’block’ of trials represents one set of the 48 unique trials: 4 targets (2 far and 2 near) x 3 load conditions (4N in +Y or -Y direction, and null-load) x 2 delay times (250 ms and 750 ms) x 2 perturbation directions (+Y and -Y). The trial setup can be seen in Table 1. In the experiment, trials were presented in a block-randomized order. This means that initially one trial was randomly selected by the script. Thereafter, another trial was randomly picked as the next trial, with the constraint that a specific trial could not be repeated until all trials of a block had been performed. Each participant performed 15 repetitions of each unique trial type, and these 720 trials took approximately 1.5 hours to finish.

**Table 1:** Trial table. There were three different load conditions, either no load was applied or a slow-rising 4N load was applied in either the +Y or–Y direction. The preparatory delay was either ’short’, i.e., 250 ms or, ’long’, i.e., 750 ms. The targets were referred to as ’1’: near target in the +Y direction, ’2’: near target in the -Y direction, ’3’: far target in the +Y direction, and ’4’: far target in the -Y direction. The perturbation was applied in either the +Y or–Y direction.

	Load (direction)	Delay (ms)	Target (id)	Perturbation (direction)
***Trial 1***	0	250	1	+Y
***Trial 2***	0	250	1	-Y
***Trial 3***	0	250	2	+Y
***Trial 4***	0	250	2	-Y
***Trial 5***	0	250	3	+Y
***Trial 6***	0	250	3	-Y
***Trial 7***	0	250	4	+Y
***Trial 8***	0	250	4	-Y
***Trial 9***	0	750	1	+Y
***Trial 10***	0	750	1	-Y
***Trial 11***	0	750	2	+Y
***Trial 12***	0	750	2	-Y
***Trial 13***	0	750	3	+Y
***Trial 14***	0	750	3	-Y
***Trial 15***	0	750	4	+Y
***Trial 16***	0	750	4	-Y
***Trial 17***	+Y	250	1	+Y
***Trial 18***	+Y	250	1	-Y
***Trial 19***	+Y	250	2	+Y
***Trial 20***	+Y	250	2	-Y
***Trial 21***	+Y	250	3	+Y
***Trial 22***	+Y	250	3	-Y
***Trial 23***	+Y	250	4	+Y
***Trial 24***	+Y	250	4	-Y
***Trial 25***	+Y	750	1	+Y
***Trial 26***	+Y	750	1	-Y
***Trial 27***	+Y	750	2	+Y
***Trial 28***	+Y	750	2	-Y
***Trial 29***	+Y	750	3	+Y
***Trial 30***	+Y	750	3	-Y
***Trial 31***	+Y	750	4	+Y
***Trial 32***	+Y	750	4	-Y
***Trial 33***	-Y	250	1	+Y
***Trial 34***	-Y	250	1	-Y
***Trial 35***	-Y	250	2	+Y
***Trial 36***	-Y	250	2	-Y
***Trial 37***	-Y	250	3	+Y
***Trial 38***	-Y	250	3	-Y
***Trial 39***	-Y	250	4	+Y
***Trial 40***	-Y	250	4	-Y
***Trial 41***	-Y	750	1	+Y
***Trial 42***	-Y	750	1	-Y
***Trial 43***	-Y	750	2	+Y
***Trial 44***	-Y	750	2	-Y
***Trial 45***	-Y	750	3	+Y
***Trial 46***	-Y	750	3	-Y
***Trial 47***	-Y	750	4	+Y
***Trial 48***	-Y	750	4	-Y

### Electromyography (EMG)

Surface electromyography (EMG) signals were recorded from seven arm muscles. The EMGs correspond to the following muscles, EMG1: *m*. *brachioradialis*, EMG2: *m*. *biceps*, EMG3: *m*. *triceps brachii caput laterale*, EMG4: *m*. *triceps brachii caput longum*, EMG5: *m*. *deltoideus pars anterior*, EMG6: *m*. *deltoideus pars posterior*, and EMG7: *m*. *pectoralis major*. The EMG electrodes (Bagnoli^TM^ DE-2.1, Delsys Inc., USA) that we used, have two silver rods with contact dimensions 10.0 x 1.0 mm and 10 mm spacing. Before the EMG electrodes were placed on the peak of the belly of the muscle, the skin was cleaned with alcohol swabs. The electrodes were coated with conductive gel. All electrodes were attached with double-sided tape and secured using surgical tape. One ground electrode (Dermatrode® HE-R Reference Electrode type 00200–3400; American Imex, Irvine, CA, USA), with a diameter of 5.08 cm, was placed on the *processus spinosus* of C7 and connected to the same EMG system (Bangoli-8 EGM System, Delsys Inc., USA). The EMG signals were pass-band filtered online through the EMG system (20–450 Hz) and sampled at 1.0 kHz.

### Data preprocessing

Data preprocessing and analysis was performed in such a way that the individual participants could not be identified. The obtained EMG data was high-pass filtered by employing a fifth-order, zero phase-lag Butterworth filter with passband 30 Hz cutoff and then rectified. Normalization was applied through z-transformation. This was done to allow EMG data from different muscles and participants to be compared. The normalization procedure (z-transformation) used here is based on the same procedure that has been described in greater detail already [36–38]. In short, first, the EMG data of each muscle is concatenated, and a grand mean and grand standard deviation are calculated for each muscle, independently. In the next step, the normalized data is obtained by subtracting the grand mean of the muscle from the measured value and dividing the difference by the grand standard deviation of the muscle. The five initial blocks of trials were viewed as a familiarization period and were removed from the subsequent data analyses. The normalized data of the remaining blocks were averaged for each participant and trial type. Data preprocessing was performed using Matlab® (MathWorks, Natick, MA, USA). The data is available at Mendeley Data, V1, doi: 10.17632/44n4m42rvw.1 [39].

### Principal component analysis (PCA)

Principal component analysis (PCA) was performed on the z-normalized EMG data. PCA [27, 40] was used to study the inherent differences in EMG activity between different experimental settings, i.e., trials. PCA is an unsupervised method used to compress multidimensional datasets by generating principal components (PCs). To evaluate the experimental design, the EMG signals were only centered as they inherently are on the same scale. Centering was applied to reduce the influence of baseline noise [27]. Centering the data means that the column mean value is subtracted from the observed value. Model evaluation was performed using cross-validation by jackknifing [41]. In this way, jackknifing was used to identify the significant number of components. The level of coherence between participants was examined using a centered PCA. Since the analysis focuses on the activity before voluntary movement, only data from -20 to +100 ms around the time of perturbation were included in the PCA. For the participant comparison, two different scaling types were used. First, the data was centered, to focus on the variables with higher values (i.e., higher EMG amplitudes). Thereafter a PCA model scale to unit variance (UV-scaling) was used to give each variable in the data set equal weight in the model. By using UV-scaling baseline noise variations are also allowed to influence the model. Contribution plots were used to visualize the degree to which a variable contributes to a specific observation [42]. Here, used to depict contributions to trials as well as participants (see Figs 4 and 6). Pearson correlation coefficients were calculated to compare the similarities between similar trials. PCA was performed using SIMCA®, version 17.0 (Sartorius, Umeå, Sweden).

## Results

To identify population trends related to the stretch reflexes, the averaged EMG activities (z) recorded from -20 ms to 100 ms around the time of perturbation were used to create a principal component analysis (PCA). Data from all participants and muscles were included. In this way, the data was focused on the EMG activity prior to voluntary movement. The PCA enabled the exploration of the dominating trends across all muscles in response to the different trial settings. PCA identified 28 significant components, where the first, second, and third components explained 22%, 13%, and 6% of the variation, respectively. The complete PCA component table is available as S1 Table.

**Fig 2:**
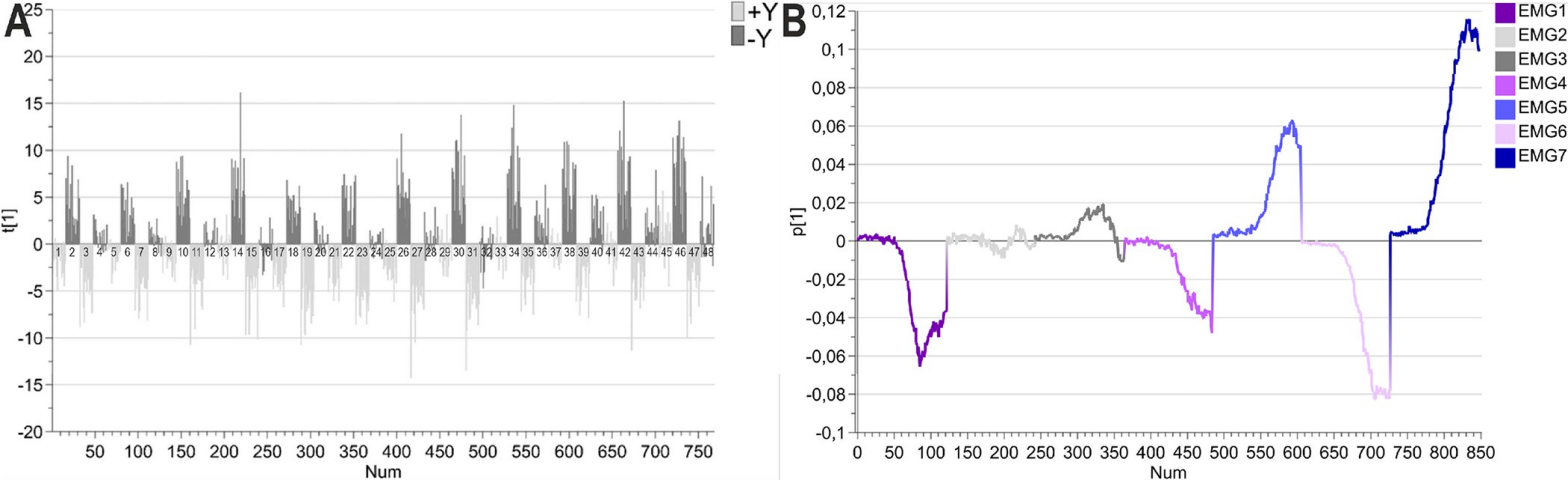
First component of the PCA. **(A) Score plot.** The normalized EMG data was centered. The first component explained 20% of the variation. Light gray indicates a +Y perturbation, Dark gray indicates -Y perturbations. Colored by perturbation. Sorted by trial (numbers indicate trial number). **(B) Loading plot.** The colors represent the different muscle functions, muscles that were triggered by a +Y perturbation were colored purple, while muscles that were triggered by a -Y perturbation were colored blue, and the muscles that responded to both were colored gray. EMG1: brachioradialis, EMG2: biceps, EMG3: triceps lateral head, EMG4: triceps long head, EMG5: anterior deltoid, EMG6: posterior deltoid, and EMG7: pectoralis.

The first component was dominated by the effect of perturbation, see Fig 2. In response to a +Y perturbation (as defined in Fig 1A), the stretch reflex was triggered in both the posterior deltoid and brachioradialis and to a certain extent also in the triceps long head. In response to a–Y load the stretch reflex was triggered in both the pectoralis and the anterior deltoid. Both the biceps and the triceps lateral head responded to both types of perturbations. The score plot (Fig 2A) and loading plot (Fig 2B) show that the stretch reflexes of the pectoralis and anterior deltoid were triggered when the cued target was in the–Y direction in combination with a–Y perturbation (i.e., dark gray positive t-values observed for trials 2, 6, 10, 14, 18, 22, 26, 30, 34, 38, 42, 46 correlate with positive p-values for EMG5 and EMG7). In addition, the plots reveal that the stretch reflexes were triggered in the posterior deltoid and the brachioradialis as well as the triceps long head when the cued target was in the +Y direction and the perturbation was applied in the +Y direction, especially when the pre-load had been applied in the +Y direction (i.e., light gray negative t-values for trials 3, 7, 11, 15, 19, 23, 27, 31, 35, 39, 43, 47 correlated with negative p-values of EMG1, EMG4, and EMG7).

**Fig 3:**
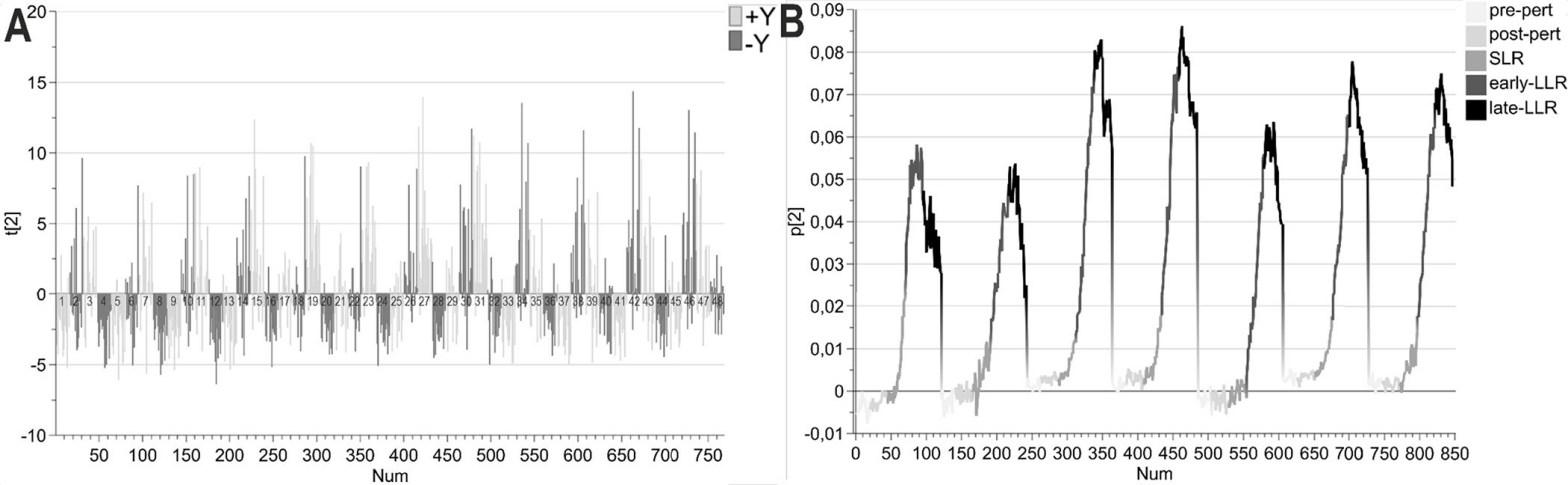
Second component of the PCA. **(A) Score plot.** The normalized EMG data was centered. The second component explained 13% of the variation. Colored by perturbation and sorted by trial (numbers indicate trial number). **(B) Loading plot.** The colors here represent the different epochs, very light gray = pre-perturbation, light gray = post-perturbation, gray = SLR epoch, dark gray = early LLR, and black = late LLR. The placement of the peaks is still the same–EMG1: brachioradialis, EMG2: biceps, EMG3: triceps lateral head, EMG4: triceps long head, EMG5: anterior deltoid, EMG6: posterior deltoid, and EMG7: pectoralis.

The second component explained the trials that triggered the highest activity in all of the EMGs, meaning that these were the participant and trial combinations that produced the strongest stretch reflex activities, see Fig 3. The score plot (Fig 3A) and loading plot (Fig 3B) shows that the stretch reflexes were not triggered when the perturbation was applied in the direction that pushed the hand into or in the direction of the target, when no load was applied (i.e., the trials where we see negative t-values). When the slow-raising 4N load was applied in the +Y direction, the stretch reflexes were not triggered when the targets were in the–Y direction (i.e., targets 2 and 4) and the perturbation took the hand into or in the direction of the target. But for the trials where targets in the +Y direction (i.e., targets 1 and 3) were cued, this gave a slight response in some of the participants, even when the perturbation took the hand into or in the direction of the target (i.e., +Y perturbation). For trials that involved a slow-raising 4N load in the–Y direction all trials that involved the perturbation took the hand into or in the direction of the target failed to trigger the stretch reflexes, except for the trials where target 4 was cued.

**Fig 4:**
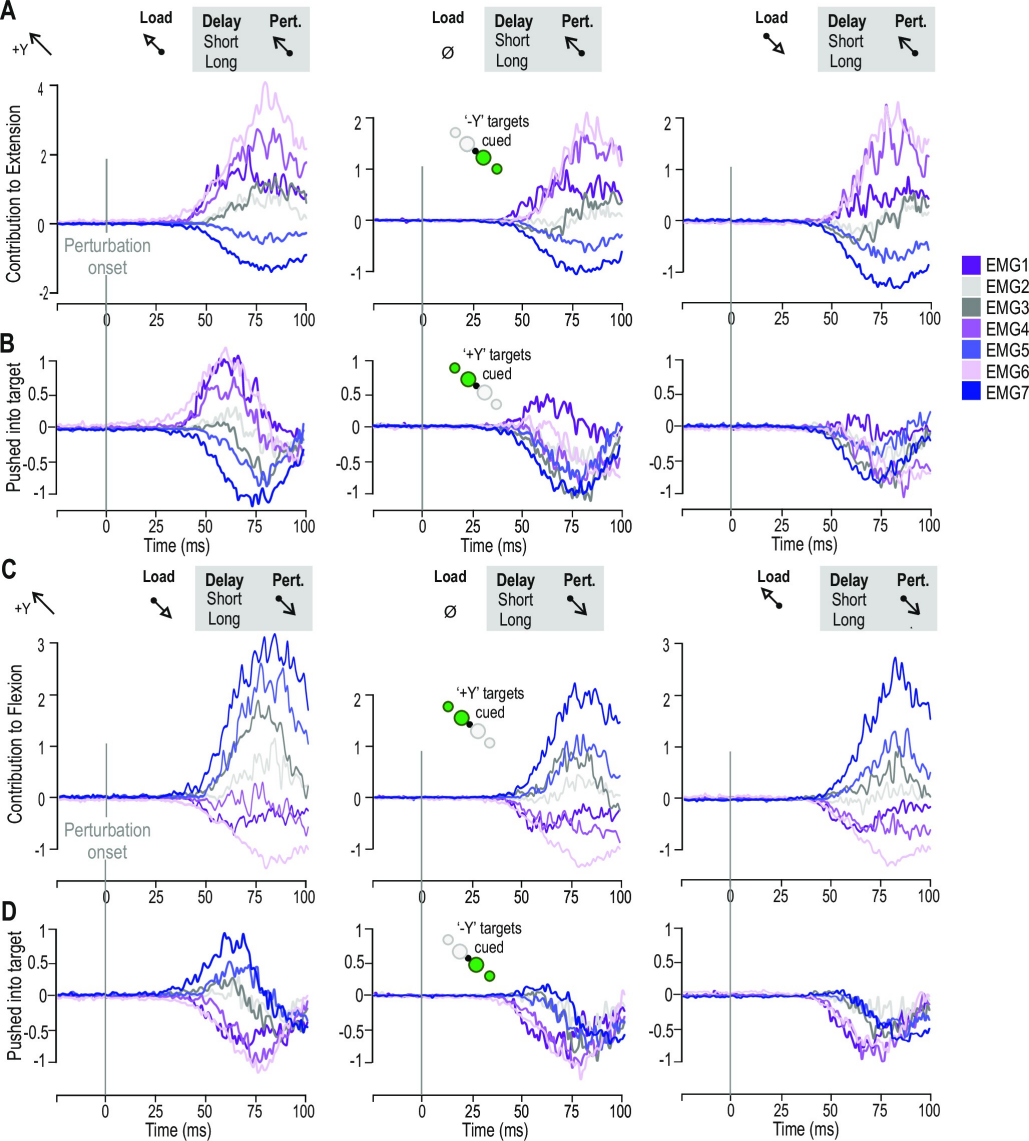
Contribution to flexion/extension overview. For clarity the data is displayed in the same timeframe instead of a long sequency. **(A)** The three different columns represent the three different loads that were applied to the left the load was applied in the -Y direction, in the middle no load was applied and to the right a +Y load was applied. For each of these trials the targets were in the -Y direction. The haptic perturbation was applied in the +Y direction. ‘Contribution to extension’ refers to contribution to shoulder extension, with an internal rotation and an elbow extension. **(B)** Loads and perturbation same as in (A), but the cued targets were in the +Y direction. ‘Pushed into target’ refers to the trials when the perturbation brought the handle into or in the direction of the target. **(C)** Loads same as (A), targets were in the +Y direction and the perturbation was applied in the -Y direction. ‘Contribution to flexion’ refers to contribution to shoulder flexion, with an internal rotation and a simultaneous shoulder extension that is required to reach the cued target. **(D)** Same loads as in (A), targets in the -Y direction and perturbation in the -Y direction, meaning that the perturbation brought the hand into or towards the cued target. The colors represent the different muscle functions, muscles with stretch reflex that were triggered by a +Y perturbation were colored purple (EMG1, EMG4 and EMG6), while muscles that were the stretch reflex was triggered by a -Y perturbation were colored blue (EMG5 and EMG7), and the muscles’ that responded to both were colored gray (EMG2 and EMG3). EMG1: brachioradialis, EMG2: biceps, EMG3: triceps lateral head, EMG4: triceps long head, EMG5: anterior deltoid, EMG6: posterior deltoid, and EMG7: pectoralis.

To study the muscle synergies, the score contribution (Trial x–Average, with weight p2) was extracted for each trial. Visual inspection of the contribution plot revealed high similarities between trials with the same perturbation, load condition, and target direction (e.g., trials 2, 6, 10, and 14). The similarities were also evaluated by Pearson correlation. The Pearson correlations when the stretch reflexes of extensors (i.e., perturbation in the +Y direction) were triggered were all ≥ 0.83 ($\bar{x}$ = 0.9, 95% CI [0.88, 0.92]), while the trials with +Y perturbation that did not trigger stretch reflexes were ≥ 0.53 ($\bar{x}$ = 0.74, 95% CI [0.67, 0.80]). The Pearson correlations when the stretch reflexes of flexors (i.e., perturbation in the -Y direction) were triggered were all ≥ 0.80 ($\bar{x}$ = 0.89, 95% CI [0.87, 0.92]), while the trials with -Y perturbation that did not trigger stretch reflexes were ≥ 0.48 ($\bar{x}$ = 0.72, 95% CI [0.63, 0.80]). Owing to the high degree of correlation between the trials that trigger the stretch reflexes, the contribution vectors were averaged across target distance and preparatory delay. The average contribution vectors were used to study how muscles contribute to the high EMG activity observed in the different trial types, based on the effect of the perturbation, see Fig 4. The difference between the two rows in Fig 4A–4D is the direction of the cued targets. By visual inspection, Fig 4A and 4B shows that the stretch reflexes of extensors were triggered the strongest when the participant had experienced a +Y 4N load and a +Y perturbation but expected to reach targets in the -Y direction. The strongest contribution to the extension came from the posterior deltoid, followed by the triceps long head, and brachioradialis. Similarly, visual inspection Fig 4C and 4D shows that the stretch reflexes of flexors were triggered the strongest when the participant had experienced a -Y 4N load and a -Y perturbation but expected to reach for targets in the +Y direction.

The principal components that follow were mainly focused on individual characteristics, where e.g., the third component explained the unique patterns associated with one participant, i.e., the low activity observed when this participant experienced a–Y perturbation, and the high activity observed in lateral triceps of this participant, compared to the other participants. Since it is already well-known that these types of naturalistic experiments will affect people in a slightly different way depending on e.g., level of training, muscle mass, stature, experience, and joint angles, the remaining component (each explaining <4% of the variation) were not included in any further analysis. Our data show that different participants display slightly different preparatory activities. For example, in some trials where one participant displayed great activity in the pectoralis, another had equivalent activity in the anterior deltoid, while a third participant used both. Similarly, some participants display high preparatory activity in the posterior deltoid, while others use the triceps long head. The data also shows that there are individual variations in how the participants handle certain factor combinations. For example, some participants displayed especially high activity in brachioradialis when being pushed in the +Y direction.

**Fig 5:**
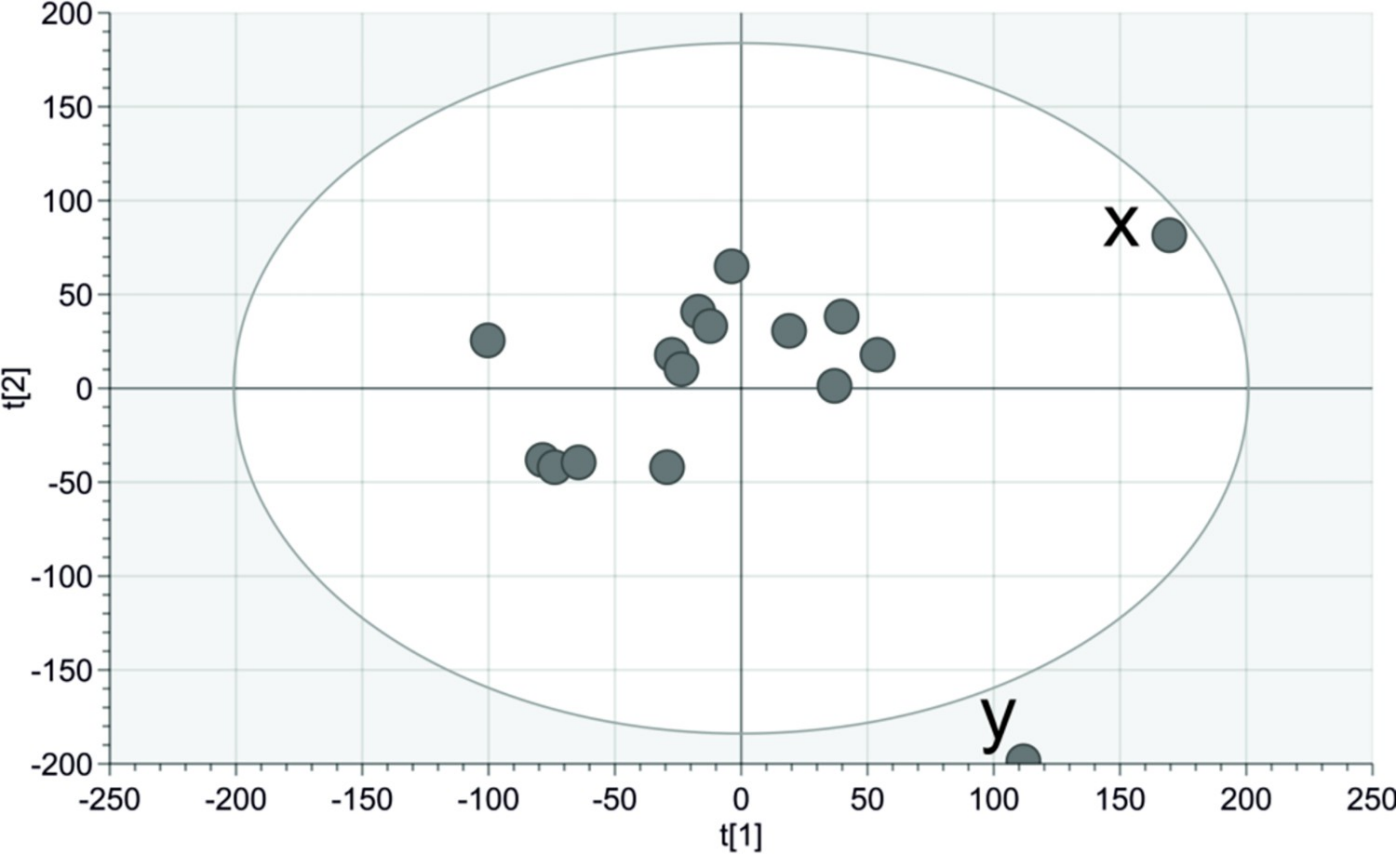
Participant overview using PCA score plot. The UV-scaled PCA had two significant components where the first and second components explained 12% and 10% of the variation respectively. The ellipse resents the 95% confidence limit for Hotelling’s T2. Fourteen of the participants end up in close proximity while two participants deviate from the group, these were named participant x and participant y.

**Fig 6:**
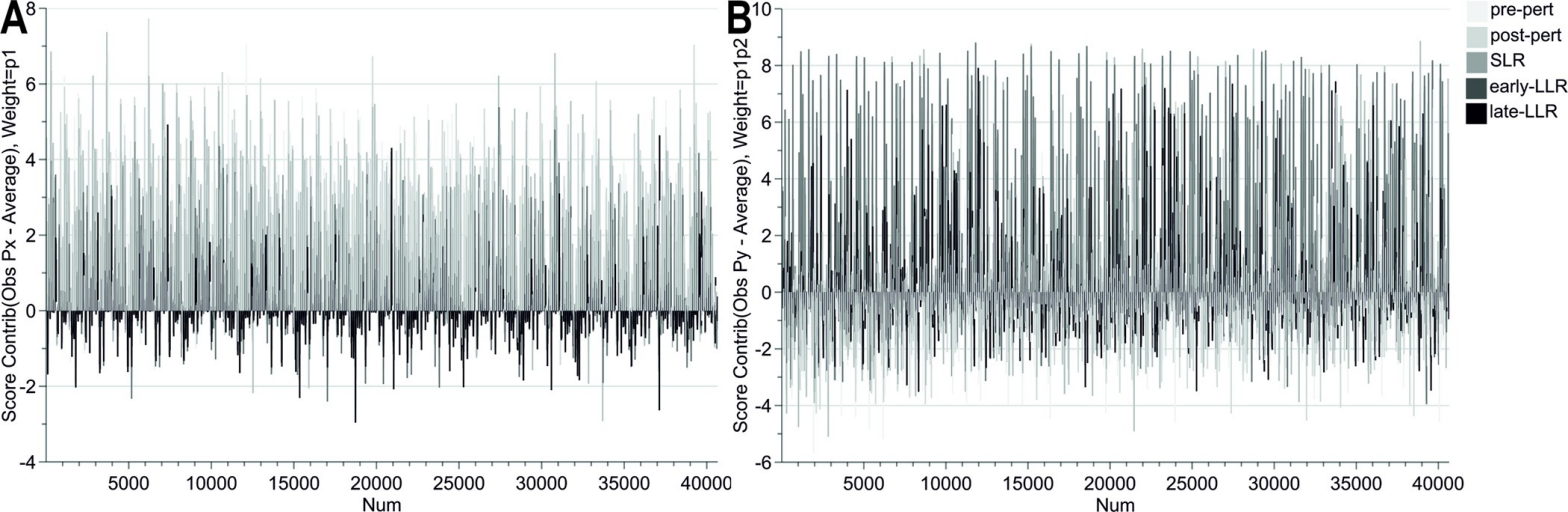
Contribution plot Participant x and y. **(A) Participant x**. The participant called x mainly deviated in the first component, hence the contribution plot only depicts the first component. **(B) Participant y.** The participant called y deviated in both the first and mainly the second component, hence the contribution plot only depicts both components. Colored by epoch. Abbreviations: Py = participant y, Px = participant x.

To evaluate the size of between-participant differences, a centered PCA was created. This resulted in zero significant components, indicating that high EMG amplitudes were observed in the participants, for the same trial conditions, to a high degree. Thereafter, a UV-scaled PCA was created, to allow the epochs with lower activity to influence the model to the same degree as the epochs with higher EMG activity (i.e., all variables are given the same weight in the model), see Fig 5. The resulting UV-scaled PCA was a two-component model where the first and second components explained 12% and 10% of the variation, respectively. The score plot clearly shows that two of the participants are diverging from the group. One of them is outside the 95% confidence limit for Hotelling’s T2. To identify the variables responsible for the deviation for the participants referred to as participant x and participant y, contributions plots were created, see Fig 6.

In general, participant x had higher EMG activity in the earlier epochs (pre-pert, post-pert, SLR) while this participant displayed a little bit lower activity in the late–LLR epoch, compared to the other participants. The main reason for this participant’s deviation from the rest of the participants was the high activity in the earlier epochs (based on amplitude). Coloring by the muscle (available as S1 Fig) revealed that the low activity in the late-LLR epoch was seen in the posterior deltoid and brachioradialis, while the high activities in the pre-perturbation and post-perturbation epochs were seen in both triceps lateral and long heads. In general, participant y had higher activity in the pre-pert epoch, but lower in the post-perturbation and SLR epochs especially after no-preload was applied, to then be higher again in both the early- and late-LLR epochs. Coloring by the muscle (available as S2 Fig) revealed that these patterns were related to high activity, regardless of target direction, in the pectoralis, and brachioradialis.

## Discussion

One of the main messages here is that the contribution plots can be used to display muscle synergies. Contribution plots display the variables that contribute the most to large score values. In this way, the contribution plot can be viewed as a link between the scores and the loadings. For the second component, the score and loading plot revealed that the large score values represented high EMG activity in the short-latency reflex (SLR), the early long-latency reflex (LLR), and the late LLR, i.e., representing trials where the stretch reflexes were triggered. The contribution plots were created to represent the individual trials, compared to the average of the model. In this way, the contribution plots were used to identify which variables have the strongest impact in the clusters representing the trials. Positive values indicate that the stretch reflex was triggered, while negative values indicate that these muscle display lower EMG activity compared to the average trial. Note that the contributions depend on summary statistics, allowing us to compare multiple studies even if different experimental designs have been used.

The muscles that were studied here were chosen on the basis that they flex or extend the elbow or shoulder joints. Based on the trial settings that were used, the movement that should take the participant’s hand to the targets in the +Y direction (as defined in Fig 1) requires flexion and internal rotation of the shoulder and extension of the elbow. This would then activate the anterior deltoid, pectoralis (clavicular fibers), triceps long head, and the triceps lateral head [1, 2, 5, 6]. The movement that should take the participant to the target in the -Y direction requires shoulder extension and elbow flexion. This would then activate the posterior deltoid, triceps long head, triceps lateral head, biceps, and brachioradialis [1, 3, 4].

The PCA (Fig 2) and the summarized contribution plots (Fig 4) showed that the combination of +Y perturbation and targets in the -Y direction activated the stretch reflexes of the posterior deltoid, triceps long head, brachioradialis, triceps lateral head, and biceps. The observed muscle activity was thereby the expected, based on the target direction. In other words, reaching for targets in the direction of muscle shortening but experiencing a perturbation away from the targets triggered high EMG amplitudes in the reflex period. The biceps was the muscle that had the lowest contribution out of the studied muscles. To increase the amplitude of the stretch reflexes of the biceps the angle of the forearm would need to be altered, as the biceps is not only a flexor but also contributes to the outwards rotation of the forearm, or that the shoulder is more externally rotated. This would not be possible owing to how the handle is manufactured (it can only be moved when the participant is gripping it with the whole hand) and would also affect the loading and unloading of the shoulder muscles. In the future, it would be interesting to include e.g., subscapularis or latissimus dorsi, either replace biceps and add both of them, or add one of them as the eighth EMG channel.

The target direction and perturbation combination decided whether the stretch reflexes were triggered or not. Similar to the goal-dependent tuning that has been reported previously [43]. The PCA (Fig 2) and the summarized contribution plots (Fig 4) also showed that the combination of -Y perturbation and target in the +Y direction triggered the stretch reflexes of the pectoralis, anterior deltoid, triceps lateral head, and biceps (for the trials with targets in the +Y direction, a -Y load, and a -Y perturbation (Fig 4C)). This was the expected outcome based on the trial settings except that the biceps should not have been triggered while the triceps long head should have been triggered as a part of the elbow extension. Others have reported shoulder stabilizing activity in the biceps [44]. This seems to be the reason for the biceps’ stretch reflex to be triggered even when for these conditions, that involve elbow extension. The triceps long head was mainly involved in the extension of the shoulder joint but could also have been activated since it contributes to the shoulder joint stability. The reason for the lack of activity in these trials is that the internal rotation of the shoulder shifts the triceps activity to the lateral head, that is loaded for the same conditions as the pectoralis and anterior deltoid (Fig 4C and 4D).

The preparatory load also shaped the size of the stretch reflex responses (Fig 4). A +Y load pre-loaded the posterior deltoid, while it unloaded the pectoralis and anterior deltoid (evident by the placement of EMG6 on top of the EMG5 and 7 in the per-perturbation and post-perturbation epochs) (left panel in Fig 4). A -Y load unloaded the posterior deltoid, while it loaded the pectoralis and anterior deltoid (evident by the placement of EMG5 and 7 on top of EMG6) (right panel in Fig 4). The amplitude of the contribution to the stretch reflexes was stronger when the muscles were loaded. It was only for a loaded muscle that the stretch reflexes were triggered even when the perturbation brought the handle into or in the direction of the cued target. This is similar to what was seen before where assistive pre-load results in higher EMG activity [43].

The Pearson correlation coefficients that were calculated for the trails that triggered the stretch reflexes and only differed in target distance or preparatory delay revealed high correlations (all Pearson correlation coefficients ≥ 0.80). Meaning that the high EMG activity occurred in a highly similar manner, independent of preparatory delay and target distance. For the trials that did not trigger the stretch reflexes the correlation was still moderate, meaning that although these trials are strongly affected by baseline variation, the Pearson correlation coefficients were ≥ 0.48. Although the contribution plots were collapsed across preparatory delay and target distance, based on high Pearson correlation coefficients, the differences in individual muscles could be of interest to explore further. However, the focus of this study was to see that the stretch reflexes were triggered in the studied muscles, not to study the stretch reflexes observed using this type of experiment, as this has been done elsewhere [43, 45].

Since surface EMG data is noisy and easily affected by tissue composition [46], it is not surprising that we identify individual quirks. Meaning that for example the proximity of the electrode and the muscle will affect the registration. Instead of having to compare the EMGs of all the participants we here present a strategy to get a quick summary of the participants. Since we know that EMG data can be very noisy, it is important to make sure that the included participants have performed the experiment in an adequate way. Obviously, we wanted to make room for the individual variation that we know is present in this type of data. This is why we chose to analyze the participants using one single PCA instead of making separate ones for each EMG. This allows a participant to display different activity in several of the muscles, without being viewed as an outlier. There must be systematic variation differences in the data for the participant to deviate in this type of model. However, it is advantageous to study each of the individual EMGs in separate PCAs as well, since this will display the variation of the individual muscles and participants. But to get a systematic overview this type of global PCA is a solid first step.

Participant y did not perform the experiment in a satisfactory way and displayed stretch reflexes that were triggered, regardless of target direction. Participant y struggled with co-contractions in the pre-perturbation epoch, not managing to relax and stay at the origin at the same time. This type of participant should be removed from any subsequent analysis. For the remaining participants, the individual quirks were viewed as variants of the norm. Indicating that they could be used to create a population average that will allow stretch reflex related study question to truly be answered. The next step is then to evaluate the grand mean of the individual EMGs and/or synergy groups. The synergy groups should not only be assumed based on the literature or the expected, but evaluated prior to awarding e.g., flexors and extensors. In this way, we ensure that the basis of the data is as reliable as possible.

The main limitations of this study were that only limited information was recorded about the participants (i.e., age, gender, and time of experiment), and that only one normalization method was used. The normalization procedure affects both the within and between participant variability [47, 48]. This type of strategy could be extended to study the effect of normalization on the within and between participant variation. The type of procedure suggested here can also be used to review kinematics data or replicates of EMG data from the same participant. In the future, it would be helpful to correlate this type of experiment with the elbow angle at origin, the angles of elbow extension/flexion and the angle of the shoulder extension/flexion at the further targets. It would also be advantageous to include other participant metadata and see how e.g., stature (height, weight, and arm length), hobbies, degree of handedness (assessed e.g., using Edinburgh Handedness Inventory), time spent to perform the experiment and how they experienced the experiment. By collecting this type of metadata we can e.g., identify factors that affect the EMG amplitude, the onset of stretch reflexes and correspond to individual quirks.

### Conclusions

In conclusion, the strategy presented here has been proven a quick and informative study evaluation tool. The contribution plots clearly display how the factor combinations that were used triggered the stretch reflexes in the studied muscles. The inclusion of biceps was found to be questionable and the other potential muscles to include could be e.g., subscapularis or latissimus dorsi. The evaluation of participant variations identified two participants as deviating from the group. The stretch reflexes of one of them, participant y, was found to be triggered regardless of target direction and struggled with co-contractions, indicating that this participant should not be included when calculating a consensus EMG profile.

## Supporting information

S1 Table PCA component summary. EMG data.
(DOCX)

S1 Fig Participant x contribution plot. Colored by muscles.
(TIF)

S2 Fig Participant y contribution plot. Colored by muscles.
(TIF)
