# Supplementary material for: Evaluation of stretch reflex synergies in the upper limb using principal component analysis (PCA)
Source: PLoS One. 2023 Oct 12;18(10):e0292807. doi: 10.1371/journal.pone.0292807 (PMC10569523; doi:10.1371/journal.pone.0292807)
Supplement: S1 Table — EMG data. (DOCX) [file pone.0292807.s001.docx]

**S1 Table PCA component summary**. EMG data.

| **Comp.** | **R2X** | **R2(cum)** | **Eigenvalue** | **Q2** | **Limit** | **Q2(cum)** | **Significance** | **Iterations** |
| --- | --- | --- | --- | --- | --- | --- | --- | --- |
| 1 | 0.22 | 0.22 | 168 | 0.21 | 0.00248 | 0.21 | R1 | 21 |
| 2 | 0.13 | 0.35 | 101 | 0.17 | 0.00248 | 0.34 | R1 | 15 |
| 3 | 0.059 | 0.41 | 45.3 | 0.086 | 0.00249 | 0.40 | R1 | 33 |
| 4 | 0.043 | 0.45 | 32.9 | 0.066 | 0.00249 | 0.44 | R1 | 39 |
| 5 | 0.033 | 0.49 | 25.4 | 0.054 | 0.00249 | 0.47 | R1 | 111 |
| 6 | 0.031 | 0.52 | 23.4 | 0.053 | 0.0025 | 0.50 | R1 | 31 |
| 7 | 0.022 | 0.54 | 16.9 | 0.038 | 0.0025 | 0.52 | R1 | 85 |
| 8 | 0.020 | 0.56 | 15.2 | 0.037 | 0.0025 | 0.53 | R1 | 96 |
| 9 | 0.018 | 0.58 | 13.7 | 0.032 | 0.0025 | 0.55 | R1 | 45 |
| 10 | 0.015 | 0.59 | 11.1 | 0.028 | 0.00251 | 0.56 | R1 | 200 |
| 11 | 0.014 | 0.61 | 11 | 0.028 | 0.00251 | 0.57 | R1 | 49 |
| 12 | 0.012 | 0.62 | 9.02 | 0.021 | 0.00251 | 0.58 | R1 | 39 |
| 13 | 0.0087 | 0.63 | 6.67 | 0.014 | 0.00252 | 0.59 | R1 | 180 |
| 14 | 0.0084 | 0.63 | 6.41 | 0.014 | 0.00252 | 0.60 | R1 | 102 |
| 15 | 0.0072 | 0.64 | 5.52 | 0.0093 | 0.00252 | 0.60 | R1 | 50 |
| 16 | 0.0060 | 0.65 | 4.64 | 0.0089 | 0.00253 | 0.60 | R1 | 200 |
| 17 | 0.0058 | 0.65 | 4.49 | 0.0077 | 0.00253 | 0.61 | R1 | 51 |
| 18 | 0.0047 | 0.66 | 3.61 | 0.0018 | 0.00253 | 0.61 | R2 | 146 |
| 19 | 0.0044 | 0.66 | 3.38 | -5.5e-06 | 0.00254 | 0.61 | R2 | 200 |
| 20 | 0.0043 | 0.67 | 3.28 | 0.00095 | 0.00254 | 0.61 | R2 | 145 |
| 21 | 0.0040 | 0.67 | 3.1 | 0.0012 | 0.00254 | 0.61 | R2 | 200 |
| 22 | 0.0040 | 0.67 | 3.03 | 0.00086 | 0.00255 | 0.61 | R2 | 200 |
| 23 | 0.0038 | 0.68 | 2.92 | 2.4e-05 | 0.00255 | 0.61 | R2 | 176 |
| 24 | 0.0036 | 0.68 | 2.77 | -0.0023 | 0.00255 | 0.61 | R5 | 200 |
| 25 | 0.0035 | 0.69 | 2.72 | -0.0010 | 0.00256 | 0.61 | R2 | 200 |
| 26 | 0.0034 | 0.69 | 2.61 | -0.0023 | 0.00256 | 0.61 | R2 | 127 |
| 27 | 0.0032 | 0.69 | 2.45 | -0.0028 | 0.00256 | 0.60 | R5 | 200 |
| 28 | 0.0032 | 0.69 | 2.44 | -0.0033 | 0.00257 | 0.60 | R2 | 200 |
