# Supplementary figures and images for: Evaluation of stretch reflex synergies in the upper limb using principal component analysis (PCA)
Source: PLoS One. 2023 Oct 12;18(10):e0292807. doi: 10.1371/journal.pone.0292807 (PMC10569523; doi:10.1371/journal.pone.0292807)

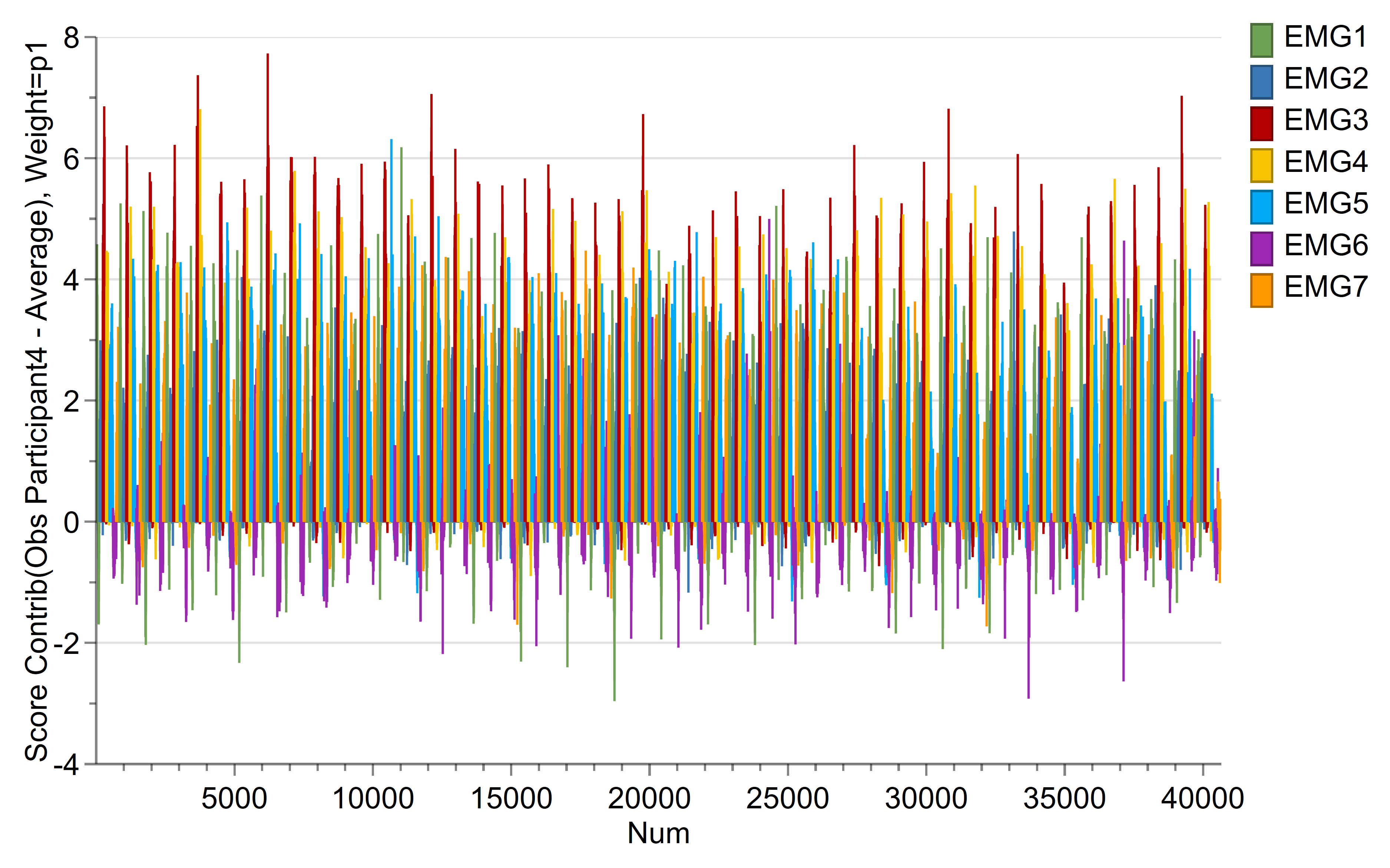

Supplement: S1 Fig — Colored by muscles. (TIF) [file pone.0292807.s002.tif]

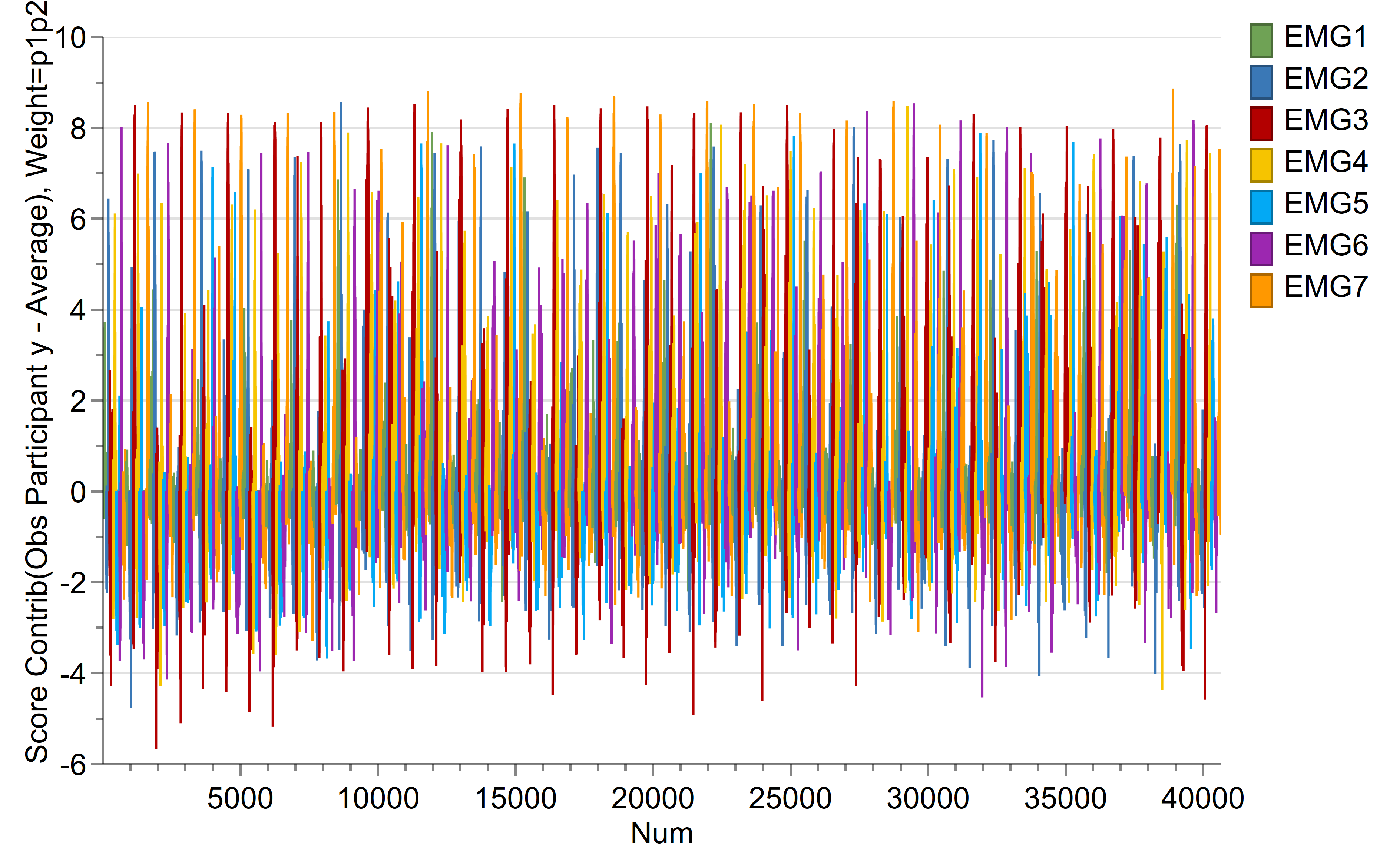

Supplement: S2 Fig — Colored by muscles. (TIF) [file pone.0292807.s003.tif]
